# Authors' reply

**Published:** 2011

**Authors:** J. Chacko, B. Gagan, E. Ashok, M. Radha, H. V. Hemanth

**Affiliations:** Multidisciplinary Intensive Care Unit, Manipal Hospital, Bangalore, India

Dear Editor,

We thank the author for showing interest in our case series of patients admitted to our multidisciplinary intensive care unit last year during the first wave of the 2009 H1N1 pandemic.[1] There are many studies, including ours, which have clearly shown that the 2009 H1N1 infection tends be more common in the previously well, relatively younger subgroup of patients.[2–5] Contrary to what the author suggests, many of our patients had underlying risk factors, although they belonged to the relatively younger age group. The median age in our series was 35 years (IQR 28.2–42.8). We sought for and identified risk factors in 64.1% of our patients – these included obesity, pregnancy, hypertension, diabetes, asthma, chronic obstructive pulmonary disease, renal failure and immunosuppression.

Prolonged ventilatory support is often required for respiratory failure from 2009 H1N1 infection. The median duration of ventilator support in our series was 10 days with an IQR of 4–22 days, which is similar to previous experience.[4,6] It is clear that severe 2009 H1N1 infection can test intensive care resources in a country like ours with serious limitation of facilities in the public sector, where the large majority of such patients are likely to be cared for.

The lung bears the brunt of the disease in 2009 H1N1 infection. The author suggests that myocardial dysfunction may be a relatively rare manifestation of 2009 H1N1 infection. However, extrapulmonary organ failure including shock is a common feature of severe 2009 H1N1 infection in the intensive care unit.[4] A significant number (58.1%) of our patients required vasopressor support – it is quite possible that the relatively high dose of analgesic and sedative drugs that we employed to facilitate mechanical ventilation could have substantially contributed to this. We did not subject our patients to systematic echocardiographic studies; however, there is evidence to suggest that subclinical cardiac dysfunction, as estimated by doppler echocardiography, may be common in these patients.[7] Rapidly progressive, fulminant myocaridits has also been reported following 2009 H1N1 infection.[8]
